# Spatial Coupling Pattern and Driving Forces of Rural Settlements and Arable Land in Alpine Canyon Region of the Maoxian County, China

**DOI:** 10.3390/ijerph20054312

**Published:** 2023-02-28

**Authors:** Lingfan Ju, Huan Yu, Qing Xiang, Wenkai Hu, Xiaoyu Xu

**Affiliations:** 1 College of Earth Sciences, Chengdu University of Technology, Chengdu 610059, China; 2 School of Earth Systems and Sustainability, Southern Illinois University Carbondale, Carbondale, IL 62901, USA; 3 Environmental Resources and Policy, Southern Illinois University Carbondale, Carbondale, IL 62901, USA

**Keywords:** rural settlements, arable land, spatial coupling, Geodetector, alpine canyon region

## Abstract

For mountainous areas in different regions, the study of the spatial coupling relationship between rural settlements and arable land resources is a key aspect of coordinated rural development. In this study, a spatial coupling relationship model and a Geodetector are introduced to explore the spatial coupling relationship and driving factors of rural settlements and arable land in the alpine canyon region. The nearest neighbor index, Voronoi diagram, and landscape pattern index system based on the geographic grid are used to analyze the spatial differentiation characteristics of rural settlements in the alpine canyon region, and the spatial coupling relationship model is introduced to explore the spatial coupling relationship between rural settlements and arable land. Finally, the driving factors of the coupling relationship are detected based on Geodetector. The results show that (1) the spatial distribution of rural settlements in the study area is “T-shaped” with a relatively regular settlement shape; (2) the population in the alpine canyon region is relatively small, and the conflict between people and land is not prominent in most areas, so the overall coupling situation between rural settlements and farming land is dominated by fewer people and more land; and (3) the spatial coupling between rural settlements and arable land in the alpine canyon region is mainly affected by four types of factors: terrain topography, meteorology, soil and population, and economy. The interaction between the factors has a synergistic enhancement effect. The results of the study provide theoretical support for the development of rural settlements in the alpine canyon region.

## 1. Introduction

Rural settlements are places where rural people engage in labor and production, carry out various social activities and live, and are platforms for communication between rural residents and the surrounding environmental elements such as geology, atmosphere, water bodies, soil, and vegetation [1]. China’s mountainous areas account for about 70% of the country’s land area. The rural settlements in mountainous areas under different geographical conditions have differences in the process of spatial evolution. This has led to an increase in the problems of disorderly settlement layout, homogeneous structure, and separation of people and land, and has become an obstacle factor in the coordinated development of mountainous areas and rural areas [2]. The pattern distribution of rural settlements and their coupling with the natural and social environment can reflect the characteristics of residents’ activities and the direction of rural development, and is an important force in promoting urban–rural integration [3]. As the most basic means of production for human beings, arable land is the material basis for the survival and development of rural inhabitants, and it presents a significant direction in the evolution of the spatial pattern of rural settlements, and the relationship between them is close and profound [4]. Therefore, in view of the characteristics of mountainous areas in different regions, the study of the coupling situation between rural settlements and arable land resources is a key link in the coordinated development of rural areas.

The change in rural characteristics and the reconstruction of sustainable rural landscapes in the context of globalization have become some of the important development directions and key research areas in international geography at present [5]. In the study of rural settlements, some scholars have focused on the relationship between rural populations, rural industries and rural settlements, the influence of public services on the morphology of rural settlements, and the evolution of rural settlement landscapes, based on the distribution forms and types of rural settlements [6]. Another part focuses on the systems and structures of rural settlements in typical areas, the geographic types of rural settlements, and the evolution of rural settlements under the influence of rapid urbanization [7,8]. In recent years, the development of rural industrialization and urbanization has led to a major change in the spatial structure and system of rural settlements, and the study of rural reconfiguration has gradually received attention [9,10].

Clarifying the spatial characteristics of rural settlements and their influencing factors is a fundamental issue in the study of the spatial pattern evolution of rural settlements. Therefore, more and more researchers have devoted themselves to the research and application of the spatial morphology of rural settlements, the relationship between settlements and surrounding appendages and the spatial reconstruction of rural settlements, and have achieved good results [11,12,13,14,15]. Currently, most studies focus on rural settlements or arable land itself [16,17,18], while studies that target the coupling dynamics between settlements and arable land are less common [19]. The spatial coupling model is a method based on grid statistics, which can better reveal the degree of coordination and the coupling situation of human–land relationships in mountainous areas [20]. The Geodetector model is a spatial analysis model that detects spatial heterogeneity and reveals the relationship between geographical attributes and their underlying influences [21]. Currently, researchers have used geographic detectors to reveal the spatial differentiation and influence factors of rural settlements or arable land, but the object of this study is only a unilateral rural settlement or arable land, without considering the two as a whole [22]. The relationship between settlements and arable land is inseparable in the development of rural revitalization in China. The Geodetector is a tool for the exploratory analysis of spatial data. It can further analyze the results of the spatial coupling model to show that each influencing factor has different degrees of influence on their spatial distribution. At the same time, it reveals the deep relationship between the coupling relationship of rural settlements and arable land and the environmental factors on spatial differentiation. This study provides a scientific basis for the evolution of rural settlements and land reconfiguration.

Therefore, this study aims specifically (i) to clarify the spatial characteristics of rural settlements in the alpine canyon region; (ii) to study the coupling dynamics between rural settlements and arable land based on a spatial coupling model; and (iii) to reveal the degree of influence of different driving factors on the coupling dynamics through a geographic detector.

## 2. Materials and Methods

### 2.1. Study Area

**Figure 1:**
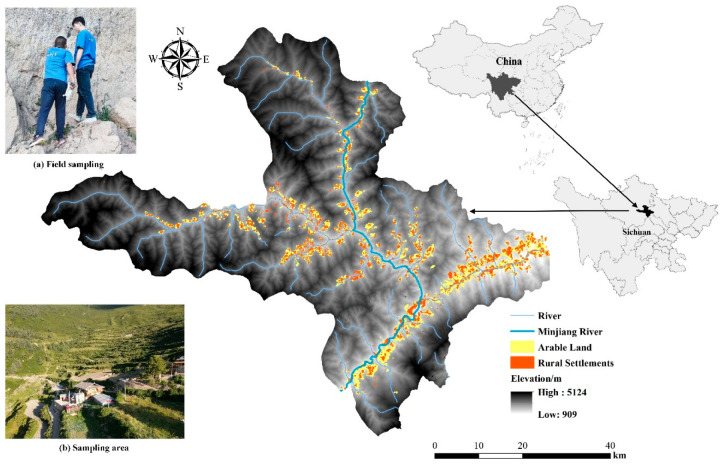
Location of the research area.

Maoxian County is located in the northwestern part of Sichuan Province and the southeastern part of Aba Tibetan and Qiang Autonomous Prefecture on the southeastern edge of the Qinghai–Tibet Plateau in China (107°48′~108°35′ E, 28°57′~29°50′ N) (Figure 1). It straddles the Minjiang River and the upper reaches of the Fujiang River, with a length of 116.62 km from east to west and a width of 93.73 km from north to south, covering an area of 3903.28 square kilometers. Maoxian County is a mountainous area with a mountain valley in the east, and the terrain is inclined from northwest to southeast, with peaks of about 4000 m in elevation and relative heights of 1500 to 2500 m. The climate of Maoxian County is influenced by the westerly wind environment and the southwest Indian Ocean monsoon, and is a plateau monsoon climate, which is characterized by a large difference in altitude, a distinct vertical climate and regional climate, and a complex local climate [23]. As of the end of 2021, the population of Mao County was 109,000, and the population of Qiang was 101,000, accounting for 92.7% of the total population [24]. It is a typical mountainous agricultural county [25], and the spatial differences between rural settlements and arable land in Mao County are significant due to the influence of topography, rivers, economic level, and ideology, so it is highly feasible and valuable to study the relationship between rural settlements and arable land in the upper Minjiang River as a research test area [26].

### 2.2. Data Sources and Processing

**Figure 2:**
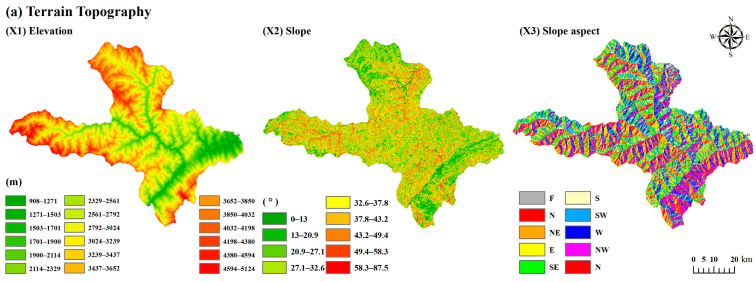

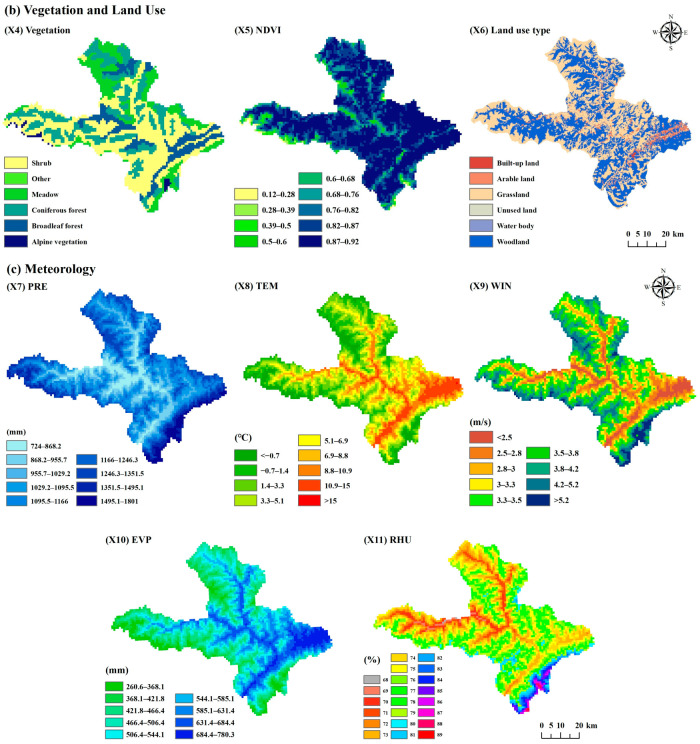

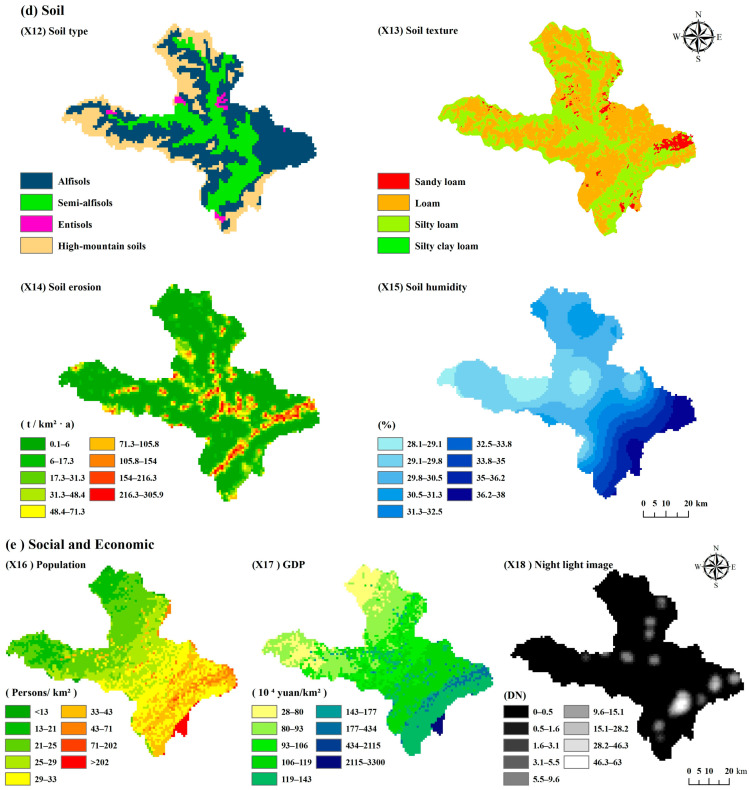
Schematic diagram of spatial distribution of terrain topography (**a**), vegetation and land use (**b**), meteorology (**c**), soil (**d**), and social and economic (**e**).

This paper uses six major categories of data, including topography and geomorphology, vegetation, land use, meteorology, soil, and socio-economics, with eighteen variable factors (Table 1): elevation, slope, land-use type, average annual wind speed, air humidity, soil type, soil texture, soil erodibility, and soil humidity data, which were obtained from National Earth System Science Data Center (http://www.geodata.cn (accessed on 14 September 2022)); vegetation type, normalized difference vegetation index (NDVI), average annual precipitation, average annual temperature, average annual evaporation, population density, and annual domestic product (GDP) data were obtained from the Resource and Environment Science and Data Center of the Chinese Academy of Sciences (http://www.resdc.cn (accessed on 30 August 2022)). Night light images were obtained from the National Centers for Environmental Information (https://www.ngdc.noaa.gov (accessed on 14 September 2022)) (Figure 2).

### 2.3. Study Methods

In this study, a 500 m × 500 m grid was used to integrate the data of land classes in the study area in order to spatially analyze the rural settlements. First, the spatial attributes data of rural settlements were assigned to the grid to which they belonged, and the spatial distribution characteristics of rural settlement locations were explored using the average nearest neighbor, Voronoi diagram, and Moran’s I. The spatial distribution characteristics of village settlement size and morphology were analyzed by Getis-Ord Gi* and landscape pattern index. Then, the spatial coupling relationship model was used to reveal the coordination degree of human–land relationship in alpine canyon region. Finally, the Geodetector model was used to explain the relationship and degree of influence of factors on the spatial coupling results.

#### 2.3.1. Average Nearest Neighbor

The average nearest neighbor (ANN) is derived from the average distance between the center of mass of each rural settlement patch and the center of mass of its nearest neighbor, and is one of the most common methods used to determine the spatial distribution pattern of rural settlements [27]. The average nearest neighbor index value is distributed between [−1, 1], the closer the result is to 1, the more discrete the distribution is, and vice versa, the more clustered it is. Therefore, the ANN is calculated as follows:

(1) $$ANN=\frac{\gamma_{\alpha }}{\gamma_{\beta }}=\frac{\sum \frac{d_{min}}{n}}{\frac{\sqrt{n/A}}{2}}=\frac{2\sqrt{\varphi }}{N}\sum d_{min}$$

In Formula (1), *γ_α_* is the average distance to the nearest neighbor of a village settlement point; *γ_β_* is the theoretical average under the spatial random distribution of village settlement points; *d_min_* is the distance between a village settlement point and its nearest neighbor; *n* is the number of village settlements; *A* is the total area of spatial units; and *φ* is the spatial distribution density of village settlements.

#### 2.3.2. Voronoi Diagram Analysis

The Voronoi diagram analysis method is commonly used to determine the spatial distribution pattern of rural settlement points and their optimization [28]. The coefficient of variation (*CV*) in the Voronoi diagram was used to further verify the distribution pattern of rural settlement sites, the formula is as follows:

(2) $$CV=\left(Std/Ave\right)\times 100\%$$

In Formula (2), Std and Ave are the standard deviation and mean of the Voronoi polygon area, respectively. When *CV* > 64%, rural settlement points are aggregated; when *CV* < 33%, rural settlement points are uniformly distributed; and when 33% < *CV* < 64%, they are randomly distributed [29].

#### 2.3.3. Landscape Pattern Index

The landscape ecology method was applied to quantitatively analyze the scale, morphology, and other characteristics of rural settlements with the help of landscape pattern index to reveal the landscape pattern of rural settlements in the Maoxian County. In this study, average patch area (AREA_MN), area-based weighted fractional dimension (FRAC_AM), landscape shape index (LSI), and average nearest neighbor Euclidean distance (ENN_MN) were selected. A total of four indicators were measured in Fragstats 4.2 platform.

#### 2.3.4. Exploratory Spatial Data Analysis

The exploratory spatial data analysis (ESDA) was used to explore the spatial distribution pattern and heterogeneity characteristics of a certain attribute of the rural settlements in the Maoxian County. In this study, Moran’s I index was selected to reflect the degree of global autocorrelation, and the Arc GIS Getis-Ord Gi* (Environmental Systems Research Institute, Redlands, CA, USA) was used to identify the distribution locations of high and low value areas with significant spatial clustering characteristics [30].

#### 2.3.5. Spatial Coupling Relationship Model

In this paper, the spatial coupling relationship model based on the grid can better reveal the coordination degree of human–land relationship in mountainous areas. In order to avoid fragmentation of the analysis results, a 500 m × 500 m grid cell is used to integrate the land class data after several experiments and comparisons. The model is as follows:

(3) $$K=S_{1}/S_{2}$$

In Formula (3), *K* is the coupling ratio between settlement and arable land; *S*_1_ is the area of settlement in unit grid; *S*_2_ is the area of arable land in unit grid, and the size of *K* value determines the coupling relationship between settlement and arable land in unit grid. When *K* < 0.04, it is “small settlement, large arable land”, there is no human–land conflict; when 0.04 < *K* < 0.28, it is “settlement and arable land coordination”, there is a balanced human–land relationship; when *K* > 0.28, it is “large settlement, small arable land”, there is a prominent human–land conflict. In addition, when there are settlements and no arable land in the cell network, it can be interpreted as the type with more people and less land, and set *K* = 88. When there are arable land and no settlements in the cell network, it can be interpreted as the type with fewer people and more land, and set *K* = 99 [9].

#### 2.3.6. The Geodetector Model

The Geodetector is an algorithm that uses the principle of spatial heterogeneity to detect driving factors of spatial coupling between rural settlements and arable land, which can quantitatively detect the influence of impact factors on spatial coupling and explore the interaction between driving factors. Geodetector includes factor detection, risk detection, interaction detection, and ecological detection [31].

First, the *q* statistic of factor detector is applied to examine the degrees proportionate to which a factor X (refers to explanatory variables of driving factors) explains the spatial heterogeneity of attribute Y (refers to spatial coupling between settlements and arable land) in an observed region. The formula is as follows:

(4) $$q=1-\frac{\sum_{h=1}^{L}N_{h}\sigma_{h}^{2}}{N\sigma^{2}}=1-\frac{SSW}{SST}$$

(5) $$\mathrm{SSW}=\sum_{h=1}^{L}N_{h}\sigma_{h}^{2}$$

(6) $$\mathrm{SST}=N\sigma^{2}$$

where *h* = 1,…, *L* is the strata of variable Y or factor X, Nh and *N* are the number of units in layer h and the whole area, respectively. $\sigma_{h}^{2}$ and $\sigma^{2}$ are the variance of variable Y in layer *h* and the whole region, respectively. *SSW* and *SST*, respectively, represent the sum of variance within the layer and the total variance of the whole region. The value of *q* can be reasoned with 0 and 1; specifically, 0 constitutes the essence of no stratified heterogeneity, whereas 1 refers to the essence of full stratification. The value of q indicates that 100 × *q*% of Y can be explained by the explanatory variable X. If the stratification is generated by the independent variable X, then a higher value of *q* implies a more robust explanatory power of the independent variable X on the attribute Y, and vice versa [32].

The effect of explanatory variables on spatially coupled outcomes may not be mutually independent. Therefore, we use an interaction detector to explore whether there is an interaction between the effects of multiple factors on the coupling. The relational, reciprocal interactions between explanatory factors can be quantified and examined via the interaction detector module. For example, the comparability between the *q*-value of the X1 and X2 factors and the interaction *q*-value of X1 ∩ X2 are analyzed as a reference to decide whether these factors enhance or weaken the interrelated dependencies or, in other cases, the factors are independent of each other. Generally, the evaluation results can be summarized into five categories (Table 2).

## 3. Results

### 3.1. Spatial Distribution Characteristics of Rural Settlements

#### 3.1.1. Cluster Distribution Characteristics Analysis

The average nearest neighbor index (ANN), Voronoi diagram analysis, and Moran’s I of the settlement patches were used to determine the spatial distribution pattern of rural settlements (Table 3). The average nearest neighbor index of rural settlements in the study area was 0.245, the CV value of the coefficient of variation in the Voronoi diagram was 578.53%, and the Moran’s I was 0.411, indicating that the spatial distribution of rural settlements in the study area was more clustered.

**Figure 3:**
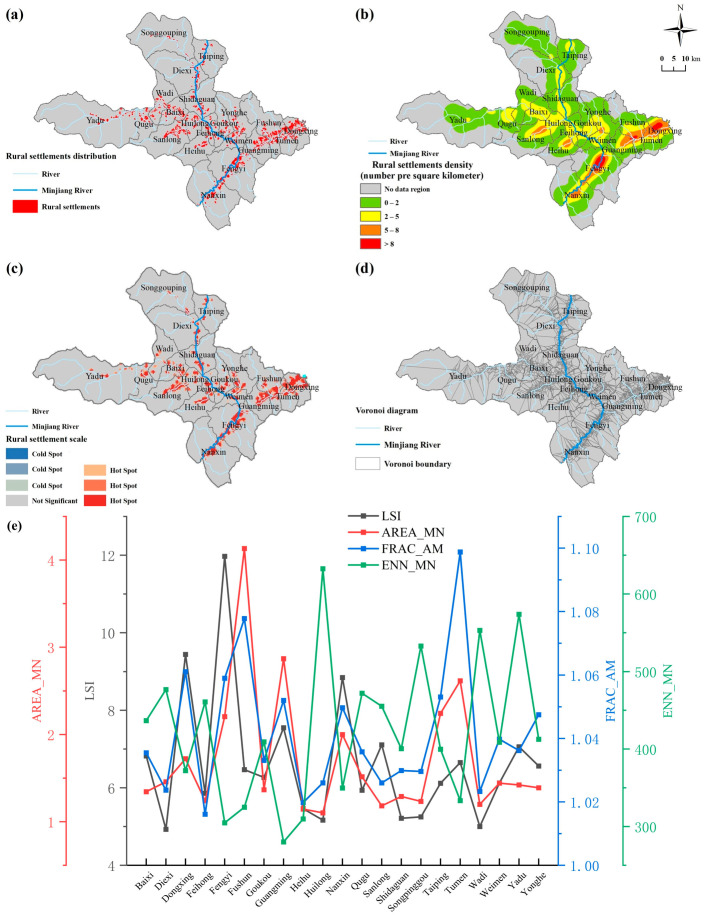
Spatial distribution of rural settlement patches (**a**), density (**b**), scale (**c**), and Voronoi (**d**). (**e**) Combination of scale and morphological indices of rural settlements at the administrative scale in the study area.

Reflecting the rural settlement patches, the density of the rural settlement patches, and the Voronoi diagram of the rural settlements in space, we discovered that the distribution of the rural settlement density in the study area showed significant clustering characteristics and obvious spatial heterogeneity (Figure 3). The geographical distribution of different settlement densities varies significantly. Settlements with densities greater than 8/km^2^ are mainly concentrated in the downstream area of the Maoxian County section of the Minjiang River (Figure 3b). This area is also the area where Feng Yi Town, the central town of Maoxian County, shows a clump-like and strip-like distribution. The settlements with lower density are mainly located in Yadu Town, which is far from the main stream of Minjiang River and at a higher altitude. According to the Voronoi diagram (Figure 3d), the areas close to the Min River and its basin water system have higher settlement densities and obvious river radiation effects. The settlement density in the eastern part of Maoxian County, which has a lower altitude, is influenced by the topography and landscape, and differs significantly from the settlement density in the northwestern part of Maoxian County and other areas.

#### 3.1.2. Scale and Morphological Characteristics

The hotspot analysis (Getis-Ord Gi*) grid number of the settlement patches was used to characterize the rural settlement scale characteristics, and the areas with more hotspot grids indicated a larger rural settlement scale (Figure 3c). The application of settlement patches based on the area-weighted fractional dimensionality (FRAC_AM) and landscape shape index (LSI) was applied to reveal the morphological characteristics of rural settlements (Figure 3e). The rural settlements in Maoxian County are characterized by large scale and clustering. Among them, the average patch area (AREA_MN) in Fushun Township is the largest, 4.13 hm^2^, and the average patch area in Huilong town is the smallest, 1.1 hm^2^; the larger and more clustered area is in Fengyi Township, whose patch area is higher than the average of the study area and has the largest number of hotspot grids. The number of sub-dimensions was similar in each township, but the landscape shape index (LSI) and the average nearest Euclidean distance (ENN_MN) were significantly different. The LSI of Fengyi town, Dongxing town, and Nanxin town are 11.97, 9.44, and 8.85, respectively, which are all higher than the average value of 6.66. This indicates that the shape of rural settlements in the region is complex. Factors such as river flow, topography, and fragmentation of arable land make the regional settlements distinctly striped. In addition, the ENN_MN of Huilong, Yadu, Songpinggou, and Wadi towns were higher and the distance between the patches was farther, indicating a longer distance between rural settlements and poorer connectivity. This is related to the spatial characteristics of “large agglomeration and small dispersion” of rural settlements within each district, as well as the wide range of administrative districts in the region.

From the spatial distribution characteristics of the village settlements, the geographical distribution characteristics of the village settlements in the study area show a “T-shaped” structure. The larger areas are mainly distributed on both sides of the Minjiang River basin and the eastern side of the lower terrain area, and are distributed in strips along the river. From the morphological characteristics of the rural settlements, the FRAC_AM of the rural settlements in each township is between 1.016 and 1.099, and the settlement pattern is relatively regular. The FRAC_AM of the west side of Minjiang River is significantly lower than that of the east side of Minjiang River, which is related to the high topography of Maoxian County in the west and low topography in the east. The average value of the landscape shape index of the rural settlements is 6.66. The overall structure of the rural settlements in the study area is not complex, and areas with a low shape index are widely distributed.

### 3.2. Coupling Situation Analysis of Rural Settlement and Arable Land

**Figure 4:**
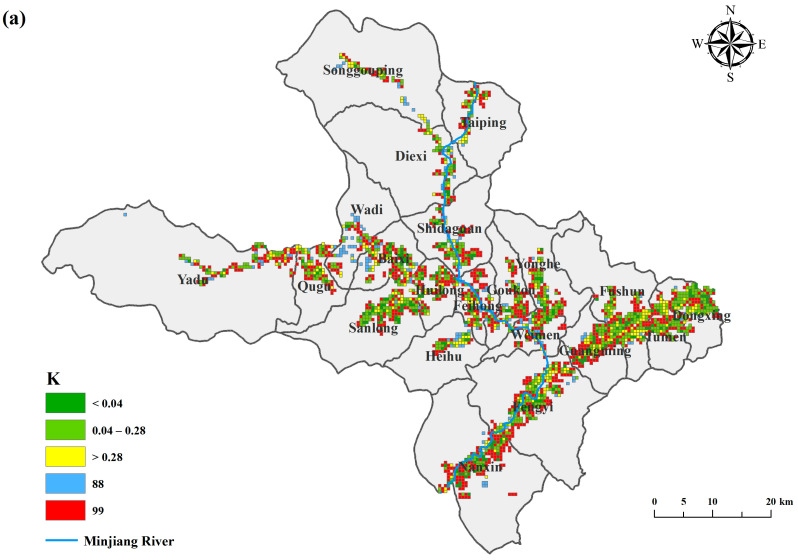

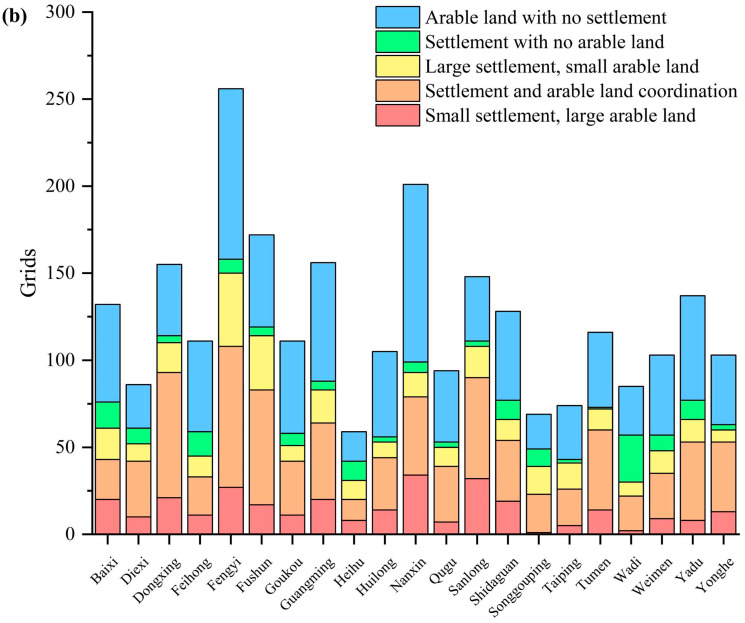
(**a**) The map of ratio of settlements area to the cropland area. (**b**) The number of coupled types of rural settlements and arable land in each township.

With the help of Arc GIS software (Environmental Systems Research Institute, Redlands, CA, USA), the coupling potential map of the rural settlement and arable land was calculated according to Formula (3) (Table 4 and Figure 4a). The results show that there are a total of 16,089 cell grids in the study area, and only 2601 grids can be calculated to obtain the coupling ratio (*K*) between the rural settlements and arable land, accounting for 16.2%. It shows that the administrative area of Mao County is vast, but the topography of the alpine canyon region affects the amount of rural settlement to arable land occupation. Among them, the highest percentage of *K* = 99 is 38.87%, followed by 0.04 ≤ *K* ≤ 0.28, which is 30.87%. ”Arable land with no settlement” and “small settlement, large arable land” belong to the type with fewer people and more land, the overall coupling between rural settlements and arable land is dominated by the type with fewer people and more land, accounting for 50.52%. The conflict between people and land in the study area is not prominent, and there are abundant arable land resources.

The central town of Fengyi and the nearby town of Nanxin have the largest number of cells in the grid, and they generally belong to the type of land with many people and few people (Figure 4b). Farming is the main economic activity of the town, with concentrated settlements, abundant arable land resources, and good coupling between the settlements and arable land. The proportion of the grid with settlements without arable land and with arable land without the settlements is 31.7% in Wadi town, and the contradiction between people and land is more prominent, and the distance between the settlements and arable land is far. Therefore, the degree of coupling between the arable land resources and rural settlements in the study area varies greatly and has obvious heterogeneity.

### 3.3. Driving Factors Analysis

#### 3.3.1. Factor Detection

In this study, the explanatory power of the q-value of the 18 impact factors and the corresponding *p*-values were obtained by the factor detection. The vegetation type, NDVI, land-use type, average annual precipitation, air humidity, soil type, and night light index failed the significance test (*p*-value less than 0.1). The remaining 11 factors had a driving effect on the spatial distribution characteristics of the spatial coupling results between the rural settlements and arable land in the study area (Table 5).

The spatial coupling of the rural settlements and arable land in the study area is driven by topographic, meteorological, soil, and social and economic factors. Among them, X9, X1, X14, and X16 are the main driving factors among the four categories of factors, with q-values of 0.0277, 0.0231, 0.0138, and 0.0121, respectively. The study area has a plateau monsoon climate, showing obvious vertical climate and regional climate phenomena with a complex local climate [33]. Among the meteorological factors, X9, X10, and X8 all have large driving factors on the coupling results, further indicating that natural factors, such as the weather in high mountain valley areas, influence the spatial coupling results of rural settlements and arable land. The landform types of the alpine canyon region mainly include river valleys and middle and high mountain landforms. It has the characteristic of “high mountains and deep valleys with little flat land”, showing a typical “V” shape [34]. The safety, livability, and future development of rural settlements are limited by the X1 (elevation). Therefore, X1 further influences the coupling outcome of rural settlements and arable land as one of the important driving factors. Soil and social and economic factors are the driving factors that directly reflect the current situation of rural settlements and arable land in the region, and in the analysis of spatial coupling results, most rural settlements and arable land are located in or around areas with higher population density and a better soil texture, in order to give full play to their potential and produce better economic and human values. The spatial coupling of rural settlements and arable land plays a certain restrictive and limiting role.

In this study, the spatial coupling between rural settlements and cropland in the study area is driven by four types of factors. Among them, X9, X1, X10, and X8 are the most important driving factors, which play a dominant role in the distribution of spatial coupling characteristics between rural settlements and arable land in the study area, thus reflecting the symbiotic relationship between rural settlements and arable land in the region, as well as the profound influence of terrain and meteorology.

#### 3.3.2. Interaction Detection

Interaction detection was performed for 11 valid factors (Table 6). It was used to reflect the size of each interaction driver in a linear and equal interval hierarchy, and it was found that the driving effects of the drivers on the spatial coupling results of rural settlements and arable land in the study area were not independent, but showed non-linear or two-factor enhancement effects. This indicates that the interaction of any 2 of the 11 drivers selected in this study has a more significant driving effect on the spatial coupling results, and that the interaction forces among the factors can better explain the geographical differences in the spatial distribution characteristics of rural settlements.

X1 ∩ X3 has the strongest influence on the coupling results between rural settlements and arable land, with the highest q-value of 0.065. This indicates that elevation and slope direction in the study area have a positive driving effect on the coupling dynamics between rural settlements and arable land. The q-values greater than 0.55 for the interactions X1 ∩ X2, X1 ∩ X10, X1 ∩ X14, X1 ∩ X15,X2 ∩ X3, X2 ∩ X9, X3 ∩ X14, and X14 ∩ X15, shows a greater effect of the two-factor than the single-factor model on the spatially coupled dynamics driving the rural settlement and arable land. Among the interactions with q-values greater than 0.25, X1 interacted most frequently, further reflecting the important role of elevation in the study area on the spatial coupling dynamics between rural settlements and arable land. Meanwhile, most of the interaction types of each factor interacting with X8, X9, X10, and X13 were bi-enhancement, while the interaction types among the remaining factors were all non-linear enhancements with more obvious enhancement effects. Meteorological and soil conditions influenced the development and layout of rural settlements and agriculture, reflecting the fundamental role on the construction and the development of rural settlements and arable land.

## 4. Discussion

In this paper, through the technical methods of landscape ecology, geography, and urban planning, a model is proposed to reflect the human–land relationship between settlements and arable land in the alpine canyon region using the spatial coupling relationship model. With the help of the Geodetector, the degree of influence and relationship of factors on the index results are analyzed. The results of the study provide a new research perspective to investigate the relationship between rural settlements and their surroundings in the alpine canyon region, and also, enhance the understanding of human settlement systems in mountainous areas.

### 4.1. Spatial Distribution Differences of Rural Settlements and Arable Land in Alpine Canyon Region

The spatial characteristics of the traditional “Mountain-Water-Forest-Field-Village” spatial settlement in alpine canyon region are more complicated due to the special characteristics of the high mountain valley terrain [35,36]. Compared with other regions, the alpine valley region is limited by the mountainous terrain, and the land in the flat dam area that is suitable for agricultural production is scarce and mostly sloping [37,38]. Therefore, the spatial distribution of the rural settlement location and morphology is convergent with that of the arable land in the study area, which strongly reflects the symbiotic relationship between people and land in the regional system of rural settlements in the alpine canyon region [18].

**Figure 5:**
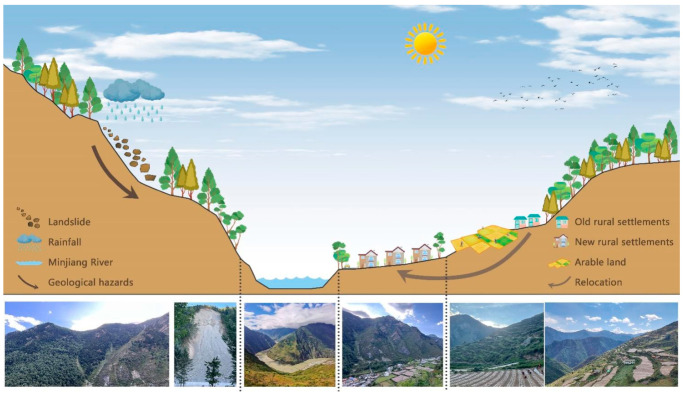
Conceptual diagram shows the geological hazards in alpine canyon region affected by weather and the separation from the original arable land caused by the relocation of rural settlements.

The research results indicate that rural settlements and arable land in Maoxian County are distributed in strips and clusters along the Min River and its tributaries. The rural settlements in the east side of Minjiang River, where the elevation is relatively low, are large in size and complex in shape. The rural settlements on the west side of the Minjiang River are mainly spatially characterized as a “large agglomeration and small dispersion”, with dense mountainous areas and a relatively lagging economy. The areas with complex morphology are more influenced by topographic conditions. The results of the study are consistent with the findings of Wang et al. and Fan et al. [1,38]. The study area is a typical territory in the alpine canyon region. Geological hazards such as landslides, slides and debris flows often accompany the study area and have a significant impact on the spatial distribution of rural settlements and arable land [39]. Therefore, rural settlements are relocated, with settlements in the higher parts of the valley being moved to the lower parts of the valley where the slope is gentler, while the original arable land is left behind in the old areas, resulting in a greater spatial separation of rural settlements and arable land [40,41] (Figure 5). At the same time, Maoxian County is a typical minority settlement [24]. The rapid economic development of the study area has brought about a large accumulation of production factors, the development of rural tourism, the development of modern leisure agriculture, the further transformation of farmers’ livelihoods, and the accessibility of rural settlements, which significantly affect the scale and morphological characteristics of rural settlements and arable land [37,42].

### 4.2. Spatial Coupling and Main Driving Factors of Rural Settlements and Arable Land

Rural China is in the process of rapid urbanization and modernization, and core factors such as rural settlements and arable land have undergone drastic changes [43]. The existence forms, functional roles, and value behaviors of rural settlements and arable land are also in a critical stage of reconfiguration and transformation [44]; therefore, in the context of rural transformation and development, it is urgent to explore the spatial coupling relationship between rural settlements and arable land to optimize the spatial pattern. The coupling ratio indicates the coupling relationship between the population and arable land in terms of quantity [45]. Most of the areas in the study area do not have prominent human–land conflicts, only the central township and the surrounding areas have prominent human–land conflicts, due to the gentler terrain and the dense layout of rural settlements with less arable land. The remaining areas have relatively less contradictory arable land in terms of quantity, but the regional topography still belongs to the mid-alpine regions [46], and the arable land is dominated by sloping arable land, and agricultural activities are greatly restricted. In the upper reaches of the Minjiang River, the Qiang and Tibetan minority populations are predominant, with low population density, relatively more arable land, and a low cluster farming ratio index.

The results of factor detection and interaction detection show that meteorological and natural factors are still antecedent to the coupling dynamics of rural settlements and arable land in the alpine valley region. Natural factors such as elevation, slope, and soil play a decisive role in the construction of early village settlements and the distribution of arable land, but as the village settlements evolve, they gradually show adaptability to the climatic environment. For example, they were selected in the flat dam areas of the river valleys, located on waterfront slopes or flat dams in well-ventilated ravines near mountain passes, and avoided hilltops and low-lying, still-wind areas where air currents were highly variable [47]. This further reveals that, based on the existing natural conditions, the spatial coupling of rural settlements and arable land in the alpine valley region is a product of the long-term use of natural resources by local people and their adaptation to the unique climate of the alpine valley region. At the same time, along with the planning and implementation of rural reconfiguration, the construction and development of future alpine valley areas will be more oriented towards serving the actual needs of people and valuing their existence [48,49]. Therefore, the influence of human factors on the spatial coupling dynamics of rural settlements and cultivated land is bound to increase.

## 5. Conclusions

This study takes the upper reaches of Minjiang River in the alpine valley region as an example and analyzes the spatial distribution characteristics of the location, scale, and form of the rural settlements using the nearest neighbor index, Voronoi diagram, and geographic grid, etc. The spatial coupling relationship between rural settlements and arable land is expressed by the coupling ratio index, and the driving factors behind the coupling situation of rural settlements and arable land in the alpine canyon region are revealed by a geographic detector. The research results show the following results:

(1) The distribution of rural residential densities in the study area shows obvious clustering characteristics and significant spatial heterogeneity. The geographical distribution of different residential densities varies greatly. Areas close to the Minjiang River and its watershed water system have higher residential densities, and the river radiation effect is obvious. The eastern part of Maoxian County is at a lower altitude, and the settlement density is influenced by the topography and geomorphology, which differs greatly from that of northwestern Maoxian County and other areas.

(2) From the spatial distribution characteristics of rural settlements, the geographic distribution characteristics of rural settlements in the study area show a “T” type structure. The larger ones are mainly distributed on both sides of the Minjiang River basin and the eastern side of the area with a lower terrain, and are distributed along the river in a belt pattern. From the morphological characteristics of the rural settlements, the distribution of the rural settlements in each township is relatively regular. The FRAC_AM on the west side of Minjiang River is significantly lower than that on the east side of the Minjiang River, which is related to the high topography in the west and low topography in the east of Mao County. The overall structure of the rural settlements in the study area is not complex, and the areas with a low shape index are widely distributed.

(3) The administrative area of the study area is vast, but the topography of the alpine canyon region affects the amount of arable land occupied by rural settlements. The overall coupling of rural settlements to arable land is dominated by the type with fewer people and more land, accounting for 50.52%. The conflict between people and land in the study area is not prominent, and there are abundant arable land resources.

(4) The spatial coupling between rural settlements and arable land in the alpine canyon region is mainly driven by four types of factors: terrain topography, meteorology, soil and population, and economy. Meteorological factors and altitude factors play a leading role, and their interactions have synergistic enhancement effects, among which the interaction of altitude factors is the most frequent and has the most prominent synergistic enhancement effect on the spatial coupling between rural settlements and arable land.

## Figures and Tables

**Table 1:** The factors that influence the spatial differentiation of rural settlements and arable land.

Factors	Sub-Factors	Code
Terrain Topography	Elevation	X1
	Slope	X2
	Slope aspect	X3
Vegetation	Vegetation type	X4
	Normalized difference vegetation index	X5
Land Use	Land-use type	X6
Meteorology	Average annual precipitation	X7
	Average annual temperature	X8
	Annual average wind speed	X9
	Average annual evaporation	X10
	Air humidity	X11
Soil	Soil type	X12
	Soil texture	X13
	Soil erodibility	X14
	Soil humidity	X15
Social and Economic	Population density	X16
	Gross domestic product	X17
	Night-time light image	X18

**Table 2:** Types of interaction between explanatory variables.

Interaction	Description
Weakened, non-linear	*q* (X1 ∩ X2) < Min (*q* (X1), *q* (X2))
Weakened, uni-factor	Min (*q* (X1), *q* (X2)) < *q* (X1 ∩ X2) < Max (*q* (X1), *q* (X2))
Enhanced, bi-factors	*q* (X1 ∩ X2) > Max (q (X1), q (X2))
Independent	*q* (X1 ∩ X2) = *q* (X1) + q (X2)
Enhanced, non-linear	*q* (X1 ∩ X2) > *q* (X1) + *q* (X2)

**Table 3:** Indicators of the spatial distribution pattern of rural settlements in the study area.

	Value	Z Score
ANN	0.245	−96.587
CV	578.53%	—
Moran’s I	0.411	73.21

**Table 4:** Integral grids with ratios of *K*.

K	Type	Grids	Ratio
<0.04	Small settlement, large arable land	303	11.65%
0.04–0.28	Settlement and arable land coordination	803	30.87%
>0.28	Large settlement, small arable land	317	12.19%
88	Settlement with no arable land	167	6.42%
99	Arable land with no settlement	1011	38.87%

**Table 5:** Results of driving factors in the study area.

Factors	Sub-Factors	Code	*q*	*p*
Terrain Topography	Elevation	X1	0.02314	0
	Slope	X2	0.01213	0.013
	Slope aspect	X3	0.00994	0.004
Vegetation	Vegetation type	X4	0.00377	0.431
	Normalized difference vegetation index	X5	0.00544	0.457
Land Use	Land-use type	X6	0.00502	0.186
Meteorology	Average annual precipitation	X7	0.01057	0.109
	Average annual temperature	X8	0.01675	0.004
	Annual average wind speed	X9	0.02775	0
	Average annual evaporation	X10	0.02064	0
	Air humidity	X11	0.02051	0.285
Soil	Soil type	X12	0.00298	0.884
	Soil texture	X13	0.00932	0
	Soil erodibility	X14	0.01384	0
	Soil humidity	X15	0.00736	0.012
Social and Economic	Population density	X16	0.01245	0
	Gross domestic product	X17	0.01108	0
	Night-time light image	X18	0.00677	0.421

**Table 6:** Interaction detection for spatial coupling between rural settlements and arable land.

	X1	X2	X3	X8	X9	X10	X13	X14	X15	X16
X2	0.059 **									
X3	0.065 **	0.057 **								
X8	0.045 **	0.044 **	0.039 **							
X9	0.053 **	0.057 **	0.048 **	0.036 *						
X10	0.056 **	0.042 **	0.047 **	0.027 *	0.039 *					
X13	0.031 *	0.024 **	0.029 **	0.025 *	0.034 *	0.027 *				
X14	0.058 **	0.051 **	0.056 **	0.043 **	0.049 **	0.049 **	0.025 *			
X15	0.064 **	0.050 **	0.046 **	0.046 **	0.053 **	0.050 **	0.018 *	0.057 **		
X16	0.046 **	0.050 **	0.042 **	0.038 **	0.043 **	0.039 **	0.021 *	0.050 **	0.038 **	
X17	0.054 **	0.042 **	0.046 **	0.045 **	0.053 **	0.046 **	0.023 **	0.051 **	0.039 **	0.032 **

Note: * and ** denote bi-enhancement and non-linear-enhancement, respectively.

## Data Availability

The data that support the findings of this study are available upon reasonable request from the authors.
